# Supplementary material for: The Sulfated PSY Peptide Negatively Regulates Receptor Kinase Activity to Promote Growth
Source: bioRxiv. 2026 Sep 2:2026.07.25.740709. Preprint. [Version 2] doi: 10.64898/2026.07.25.740709 (PMC13618415; doi:10.64898/2026.07.25.740709)
Supplement: Supplementary file 1 [file media-1.pdf]

# A

A. thaliana

AtPSYR1,2,3(At1g17230, At2g33170, At5g63930) confirmed

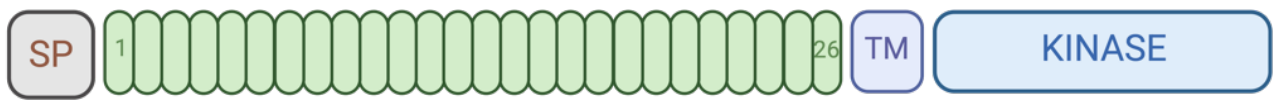


P. Patens

PpPSYR1(Pp3c24_15950) and PpPSYR2(Pp3c8_17530) putative orthologs

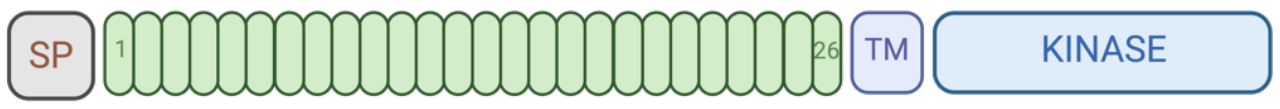


O. sativa*

OsPSYR1(LOC_Os07g05740)and OsPSYR2(LOC_Os04g42700)putative orthologs

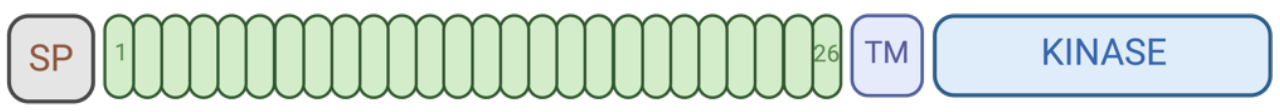


Signal Peptide | LRR Repeat | Transmembrane Domain | Kinase Domain

*Putative rice PSYreceptors:
OsPSYR1(LOC_Os07g05740)- 57.7% identity to AtPSYR1, 51.24% to AtPSYR3,51.04% to AtPSYR2

OsPSYR2(LOC_Os04g42700)-63.85% identity to AtPSYR3, 61.85% to AtPSYR2,53.67% to AtPSYR1

**Reciprocal Best Hit Analysis:
NCBI BlastP against A. thaliana set, taxid: 3702

## OsPSYR1(LOC_Os07g05740)

```
MAASVARVLLAAAVFFAAVAAAAA      1...24   signal
ASSSAAAVAALMEFKTKLDDVDGRLS   25...50
SWDAAGGSGGGDPCGWPGIACSAAME   51...76   LRRNT_2

Lx-xLxLxxNxLSGxIPxxLGxLxx     consensus
:   : :  : :::  ::  :: :
VT-AVTLHGLNLHGELSAAVCALPR     77..100  LRR 01
LA-VLNVSKNALAGALPPGLAACRA    101..124  LRR 02
LE-VLDLSTNSLHGGIPPSLCSLPS    125..148  LRR 03
LR-QLFLSENFLSGEIPAAIGNLTA    149..172  LRR 04
LE-ELEIYSNNLTGGIPTTIAALQR    173..196  LRR 05
LR-IIRAGLNDLSGPIPVEISACAS    197..220  LRR 06
LA-VLGLAQNNLAGELPGELSRLKN    221..244  LRR 07
LT-TLILWQNALSGEIPPELGDIPS    245..268  LRR 08
LE-MLALNDNAFTGGVPRELGALPS    269..292  LRR 09
LA-KLYIYRNQLDGTIPRELGDLQS    293..316  LRR 10
AV-EIDLSENKLTGVIPGELGRIPT    317..340  LRR 11
LR-LLYLFENRLQGSIPPELGELTV    341..364  LRR 12
IR-RIDLSINNLTGTIPMEFQNLTD    365..388  LRR 13
LE-YLQLFDNQIHGVIPPMLGAGSN    389..412  LRR 14
LS-VLDLSDNRLTGSIPPHLCKFQK    413..436  LRR 15
LI-FLSLGSNRLTGNIPPGVKACRT    437..460  LRR 16
LT-QLQLGGNMLTGSLPVELSLLRN    461..484  LRR 17
LS-SLDMNRNRFSGPIPPEIGKFRS    485..508  LRR 18
IE-RLILSENYFVGQIPPGIGNLTK    509..532  LRR 19
LV-AFNISSNQLTGPIPRELARCTK    533..556  LRR 20
LQ-RLDLSKNSLTGVIPQELGTLVN    557..580  LRR 21
LE-QLKLSDNSLNGTVPSSFGGLSR    581..604  LRR 22
LT-ELQMGGNRLSGQLPVELGQLTA    605..628  LRR 23
LQIALNVSYNMLSGEIPTQLGNLHM    629..653  LRR 24
LE-FLYLNNNELEGEVPSSFGELSS    654..677  LRR 25
LL-ECNLSYNNLAGPLPSTTLFQHM    678..701  LRR 26

DSSNFLGNNGLCGIKGKSCSGLSGS    702..726  LRRCT
AYASREAAVQKKRLLREK           727..744
IISISSIVIAFVSLVLIAVVCWSL     745..768  transmemb.
```

# B

## AtRGI1/AtRGFR1(At4g26540)

```
MPPNIYRLSFFSSLLCFFFIPCFS      1...24   signal
LDQQGQALLSWKSQLNISGDAFSS     25...48
WHVADTSPCNWVGVKCNRRGE        49...69   LRRNT_2

LxxLxLxxNx-LSGxIPxxLGxLxx     consensus
:  : :  : -:::  ::  :: :
VSEIQLKGMD-LQGSLPVTSLRSLKS    70...94  LRR 01
LTSLTLSSLN-LTGVIPKEIGDFTE     95..118  LRR 02
LELLDLSDNS-LSGDIPVEIFRLKK    119..142  LRR 03
LKTLSLNTNN-LEGHIPMEIGNLSG    143..166  LRR 04
LVELMLFDNK-LSGEIPRSIGELKN    167..190  LRR 05
LQVLRAGGNKNLRGELPWEIGNCEN    191..215  LRR 06
LVMLGLAETS-LSGKLPASIGNLKR    216..239  LRR 07
VQTIAIYTSL-LSGPIPDEIGYCTE    240..263  LRR 08
LQNLYLYQNS-ISGSIPTTIGGLKK    264..287  LRR 09
LQSLLLWQNN-LVGKIPTELGNCPE    288..311  LRR 10
LWLIDFSENL-LTGTIPRSFGKLEN    312..335  LRR 11
LQELQLSVNQ-ISGTIPEELTNCTK    336..359  LRR 12
LTHLEIDNNL-ITGEIPSLMSNLRS    360..383  LRR 13
LTMFFAWQNK-LTGNIPQSLSQCRE    384..407  LRR 14
LQAIDLSYNS-LSGSIPKEIFGLRN    408..431  LRR 15
LTKLLLLSND-LSGFIPPDIGNCTN    432..455  LRR 16
LYRLRLNGNR-LAGSIPSEIGNLKN    456..479  LRR 17
LNFVDISENR-LVGSIPPAISGCES    480..503  LRR 18
LEFIDLHTNS-LSGSLLG-TTLPKS    504..526  LRR 19
LKFIDFSDNA-LSSTLPPGIGLLTE    527..550  LRR 20
LTKLNLAKNR-LSGEIPREISTCRS    551..574  LRR 21
LQLLNLGEND-FSGEIPDELGQIPSL   575..599  LRR 22
AISLNLSCNR-FVGEIPSRFSDLKN    600..623  LRR 23
LGVLDVSHNQ-LTGNLNV-LTDLQN    624..646  LRR 24
LVSLNISYND-FSGDLPN-TPFFRR    647..669  LRR 25

LPLSDLASNRGLYISNA            670..686
ISTRPDPTTRNSSVVR             687..702
LTILILVVVTAVLVLMAVYTLV       703..724  transmemb.
```

```
           LRR
AtPSYR1    05    LQELVIYSNNLTGVIPPSMAKLRQ
AtPSYR2    05    LEELVAYTNNLTGPLPRSLGNLNK
AtPSYR3    05    LSQLVTYSNNISGQLPRSIGNLKR
PpPSYR1    05    LEELLCYTNNLTGPLPASLGNLKH
PpPSYR2    05    LQELLCYTNNLTGPLPASLGDLKE
OsPSYR1    05    LEELEIYSNNLTGGIPTTIAALQR
OsPSYR2    05    LEDLVGYSNNLSGSIPHTIGRLKN

AtPSYR1    06    LRIIRAGRNGFSGVIPSEISGCES
AtPSYR2    06    LTTFRAGQNDFSGNIPTEIGKCLN
AtPSYR3    06    LTSFRAGQNMISGSLPSEIGGCES
PpPSYR1    06    LRTIRAGQNAIGGPIPVELVGCEN
PpPSYR2    06    LRYIRAGQNVIGGPIPVEISNCTN
OsPSYR1    06    LRIIRAGLNDLSGPIPVEISACAS
OsPSYR2    06    LKTVRLGQNAISGNIPVEIGECLN

AtPSYR1    07    LKVIGLAENLLEGSLPKQLEKLQN
AtPSYR2    07    LKLIGLAQNFISGELPKEIGMLVK
AtPSYR3    07    LVMIGLAQNQLSGELPKEIGMLKK
PpPSYR1    07    LMFFGFAQNKLTGGIPPQLGRLKN
PpPSYR2    07    LLFIGFAQNKLTGIIPPQLSLLTN
OsPSYR1    07    LAVIGLAQNNLAGELPGELSRLKN
OsPSYR2    07    LVVFGLAQNKLGGPLPKEIGKLTN
```

LRR05 Y=RGFRAsp-174
LRR06 R=RGFRArg-195
LRR06 G= RGFRGly-197
LRR07 G= RGFRGly-220
LRR07 A= RGFRAla-222
(Songet al., 2016)

**Figure S1**
