# Supplementary material for: The Sulfated PSY Peptide Negatively Regulates Receptor Kinase Activity to Promote Growth
Source: bioRxiv. 2026 Sep 2:2026.07.25.740709. Preprint. [Version 2] doi: 10.64898/2026.07.25.740709 (PMC13618415; doi:10.64898/2026.07.25.740709)
Supplement: Supplementary file 2 [file media-10.pdf]

Table S1. List of mutant lines used and created.

Name	Background	Genetic alteration	Translation
Wild type	Gransden 2004 (*67*)	N/A	N/A
Δtpst (*26*)	Wild type	7 bp deletion	Stop at AA90, aa 81-89 are irrelevant
Δpsyr1/2	Wild type	*PSYR1:* 9 bp deletion, 4 bp insertion	*PSYR1:* Stop at AA41, aa 40 is irrelevant, deletion of AA 27-29
		PSYR2: 1 bp insertion, 1 bp change, 18 bp deletion	*PSYR2*: Stop at AA105, silent mutation in aa 104
Δpsyr1/2/Δtpst	*Δpsyr1/2*	Stop oligo insertion	Stop at AA85, aa 82-84 are irrelevant
PSYR1-mScarlet	Wild type	Insertion of the sequence encoding for mScarlet at the 3' end of the *PSYR1* gene at the endogenous locus	C-terminal fusion of PSYR1 with mScarlet
Δtpst/PSYR1-mScarlet	*Δtpst*	Insertion of the sequence encoding for mScarlet at the 3' end of the *PSYR1* gene at the endogenous locus	C-terminal fusion of PSYR1 with mScarlet
PSYR2-mScarlet	Wild type	Insertion of the sequence encoding for mScarlet at the 3' end of the *PSYR2* gene at the endogenous locus	C-terminal fusion of PSYR2 with mScarlet
Δtpst/PSYR2-mScarlet	*Δtpst*	Insertion of the sequence encoding for mScarlet at the 3' end of the *PSYR2* gene at the endogenous locus	C-terminal fusion of PSYR2 with mScarlet
psyr1-K843E/Δpsyr2 (*psyr1-KD*)	*PSYR1-mScarlet*	*PSYR1:* A5109G (AA change), C5090A(silent), T5093A (silent), G5096A (silent), C5097T (silent), A5099G (silent), A5102T (silent), G5105T (silent) *PSYR2:* 1 bp change, 10 bp deletion	*PSYR1:* K841E (kinase inactive) *PSYR2*: Stop at AA118, aa 104-117 are irrelevant
Δtpst/psyr1-K843E/Δpsyr2 (*Δtpst/psyr1-KD*)	*Δtpst/PSYR1-mScarlet*	*PSYR1:* A5109G (AA change), C5090A(silent), T5093A (silent), G5096A (silent), C5097T (silent), A5099G (silent), A5102T (silent), G5105T (silent) *PSYR2*: 7 bp deletion	*PSYR1:* K841E (kinase inactive) *PSYR2*: Stop at AA117, aa 109-116 are irrelevant

ACT1::PSYR1-mStayGold #22 and #23 (*PSYR1-OE #22 and #23*)	*PSYR1-mScarlet*	Insertion of the sequence encoding for mStayGold at the 3' end of the *PSYR1* CDS targeted to the pTK locus	Overexpression of C-terminal fusion of PSYR1 with mStayGold
ACT1::PSYR2-mStayGold #9 (*PSYR2-OE*)	*PSYR1-mScarlet*	Insertion of the sequence encoding for mStayGold at the 3' end of the *PSYR2* CDS targeted to the pTK locus	Overexpression of C-terminal fusion of PSYR2 with mStayGold
PSYR1-OE #22/psyr1-K843E	*PSYR1-OE #22*	A5109G (AA change), C5090A(silent), T5093A (silent), G5096A (silent), C5097T (silent), A5099G (silent), A5102T (silent), G5105T (silent)	K843E (kinase inactive)
PSYR2-OE /psyr2-K841E	*PSYR2-OE*	A5518G (AA change), T5502A (silent), G5505A (silent), C5508G (silent), A5511C (silent), G5514T (silent),	K841E (kinase inactive)
