# Supplementary material for: The Sulfated PSY Peptide Negatively Regulates Receptor Kinase Activity to Promote Growth
Source: bioRxiv. 2026 Sep 2:2026.07.25.740709. Preprint. [Version 2] doi: 10.64898/2026.07.25.740709 (PMC13618415; doi:10.64898/2026.07.25.740709)
Supplement: Supplementary file 3 [file media-12.pdf]

Table S3. Table of oligos

Name	Sequence (5' to 3')	Purpose
PSYR1-PS1	ATAGGAAGGTGGAGGACTTACAACCCATGGCTCTCGCCGTGAGCTTAGgttttagagctatgctgaaaag	PSYR1 protospacer
PSYR1-gt-F	GGAGTGGTTTGTCGACGAAGG	PSYR1 genotyping
PSYR1-gt-R	CTGACCAGCTCGAATTGTCCG	PSYR1 genotyping
PSYR1-gt-R3	GACAGCAACGCCACTAAGC	PSYR1 genotyping, anneals to protospacer 1
PSYR2 PS2	ATAGGAAGGTGGAGGACTTACAACCCATACGACAGGTTAAGATACCGGgttttagagctatgctgaaaag	PSYR2 protospacer
PSYR2-gt-F1	CATAGCTCGGCATGAGGAGG	PSYR2 genotyping
PSYR2-gt-R2	GGAGGTTAGGTTGCCGAGAC	PSYR2 genotyping
PSYR2-gt-R1	ACGACAGGTTAAGATACCGGAGG	PSYR2 genotyping, anneals to protospacer 1
TPST protospacer 1	ATAGGAAGGTGGAGGACTTACAACCCATatattcctcgaaccggcggcGTTTTAGAGCTATGCTGAAAAG	TPST protospacer
TPST stop oligo F	ggaaaattctggaacttctgttctttttgcatattcctcgaaTAGCTGATTAAacttatcaccagtggtatggatttact	Stop oligo for TPST
TPST stop oligo R	agtaaatccataccactggtgataagtTTAATCAGCTAttcgaggaatatgcaaaaagaacagaagttccagaattttcc	Stop oligo for TPST
TPST-comp-F	ttctctcttgtctctgttgatcacc	TPST genotyping
TPST-cutsiteF	atattcctcgaaccggcgg	TPST genotyping, anneals to protospacer 1
TPST-compR	gcgcaacagtctacgtgc	TPST genotyping

PSYR1_5arm_F	ACAAGTTTGTACAAAAAAGCAGGCTCATTCATCGCGACATCAAGTCCAAC	PSYR1-mScarlet construct preparation
PSYR1_5arm_R	tgctcaccatcgatcctccgccaccTGGTTTATCAGAGTTGGTCTTCCCAG	PSYR1-mScarlet construct preparation
PSYR1_3arm_F	cggcatggacgagctgtacaagTGAAGGGCAAGGCAGGAATGG	PSYR1-mScarlet construct preparation
PSYR1_3arm_R	ACCACTTTGTACAAGAAAGCTGGGCCATCAACATCCATCCATACCTGAAA	PSYR1-mScarlet construct preparation
PSYR2_5arm_F	ACAAGTTTGTACAAAAAAGCAGGCTGTATGATTACATGCCGAAGGGGA	PSYR2-mScarlet construct preparation
PSYR2_5arm_R	cttgctcaccatcgatcctccgccaccAGGCTTGTCAGAATTCTCAGAAATGATC	PSYR2-mScarlet construct preparation
PSYR2_3arm_F	cggcatggacgagctgtacaagTAGCTCGGCAGGAAAGGGCTACT	PSYR2-mScarlet construct preparation
PSYR2_3arm_R	ACCACTTTGTACAAGAAAGCTGGGGCGCACAACTACTCGAGTACC	PSYR2-mScarlet construct preparation
GGGS-FP-F	ggtggcggaggatcgatggtgagcaagggcgagg	GGGS linker sequence for mScarlet
FP-no stop-R	cttgtacagctcgtccatgcc	mScarlet amplification
PSYR1 PS2	ATAGGAAGGTGGAGGACTTACAACCCATGCCTTGCCCTTGTACCAGGGgttttagagctatgctgaaaag	PSYR1 c-terminus tagging protospacer
PSYR2 PS2	ATAGGAAGGTGGAGGACTTACAACCCATCCTGCCGAGTGATTGTACCAgttttagagctatgctgaaaag	PSYR2 c-terminus tagging protospacer
PSYR1-gt-F2	CCGCCACCGAAAATTTCAGTGAT	PSYR1-genotyping
PSYR1-gt-R3	ACCGCTGTGTGAGTACGTAACA	PSYR1-genotyping
PSYR2-gt-F2	CTGGCGCTACACAAGTAGCAAG	PSYR2-genotyping
PSYR2-gt-R3	TGTTGCTGTAAACGTATACCCGGA	PSYR2-genotyping
PSYR1-ps3	ATAGGAAGGTGGAGGACTTACAACCCATACCGCTATTAGCTGACCGCCgttttagagctatgctgaaaag	PSYR1 protospacer for PSYR1-K843E

PSYR1-ps4	ATAGGAAGGTGGAGGACTTACAACCCATGCCTGGCGGTCAGCTAATAGgttttagagctatgctgaaaag	PSYR1 protospacer for PSYR1-K843E
PSYR1_K843E_oligo-F	GCATGTGGGACAGTGTACAAAGCGCAAATGCCTGGaGGaCAattgATtGCtGTCgAGAAGGTTGCGACTCATCTAGACTCAGGTTTGACT	PSYR1 kinase dead oligo
PSYR1_K843E_oligo-R	AGTCAAACCTGAGTCTAGATGAGTCGCAACCTTCTcGACaGCaATcaatTGtCCtCCAGGCATTTGCGCTTTGTACACTGTCCCACATGC	PSYR1 kinase dead oligo
PSYR1-K843E-gt-F1	CCAGTTCCAGTAGCATGTCCAC	PSYR1 genotyping
PSYR1-K843E-gt-R1	TGACCGCTATTAGCTGACCG	PSYR1 genotyping, anneals to protospacer
PSYR1-K843E-gt-R2	ACCCTGCTATTGCAGACATCG	PSYR1 genotyping
PSYR2-ps2	ATAGGAAGGTGGAGGACTTACAACCCATGGTGAGTGGTCAGGTCATAGgttttagagctatgctgaaaag	PSYR2 protospacer for PSYR2-K841E
PSYR2_K841E_oligo-F	AGTGGAACAGTCTACAAAGCTGTGATGGTGAGtGGaCAaGTgATcGCtGTTgAGAAGATGTCCACTCAGACAGAATCAGGCTTGACTCAG	PSYR2 kinase dead oligo
PSYR2-K841E_oligo-R	CTGAGTCAAGCCTGATTCTGTCTGAGTGGACATCTTCTcAACaGCgATcACtTGtCCaCTCACCATCACAGCTTTGTAGACTGTTCCACT	PSYR2 kinase dead oligo
PSYR2-K841E-gt-F	GTGGTCAGGTCATAGCGGTTAAG	PSYR2 genotyping, anneals to protospacer
PSYR2-K841E-gt-R	CACAGCGATCTTATATCGCAGGTC	PSYR2 genotyping
PSYR2-F2	CTGGCGCTACACAAGTAGCAAG	PSYR2 genotyping
attB1-F	ACAAGTTTgtacaaaaaagcaggct	Linearize pTK-UBI plasmid around Ubi fragment
pTK-5arm-R	TCGACTTCCCTGCTTCGATCC	Linearize pTK-UBI plasmid around Ubi fragment
Act1pro-F	GGATCGAAGCAGGGAAGTCGAtcgaggtcattcatatgcttgagaagag	Act1 promoter amplification for pTK-Act1
Act1pro-R	agcctgctttttgtacAAACTTGTvggatcctctagatcttctacctacaaaaaagc	Act1 promoter amplification for pTK-Act1

PSYR1-cDNA-F	ATGACGAGGGGGAGGGAGAC	PSYR1 cDNA amplication
PSYR1-cDNA-R	TGGTTTATCAGAGTTGGTCTTCCCAG	PSYR1 cDNA amplication
attB1-PSYR1-F	ACAAGTTTgtacaaaaaagcaggctATGACGAGGGGGAGGGAGAC	PSYR1 fragment for pTK-Act1
PSYR1-linker-R	tcctgaggctcccgatgctccTGGTTTATCAGAGTTGGTCTTCCCAG	PSYR1 fragment for pTK-Act1
linker-mStayGold	ggagcatcgggagcctcaggagcatcgatggtgtctacaggcgaggag	mStayGold fragment for pTK-Act1
mStayGold-attB2-R	ACCACTTTGTACAAGAAAGCTGGGTttacaggtgggcctccagggtctc	mStayGold fragment for pTK-Act1
attB1-PSYR2-F	ACAAGTTTgtacaaaaaagcaggctATGAGGAGGAGAGGGGCGTGG	PSYR2 fragment for pTK-Act1
PSYR2-linker-R	tcctgaggctcccgatgctccAGGCTTGTCAGAATTCTCAGAAATGATC	PSYR2 fragment for pTK-Act1
mStayGold-R2	ctggtgcacacgtacttgcc	PSYR1 or PSYR2-mSG genotyping
EF1alpha-RT-F2	CCCTTCAGGATGTCTACAAGATT	RT-RT-qPCR primer
EF1alpha-RT-R2	CCCTTCAGGATGTCTACAAGATT	RT-qPCR primer
PSYR1-RT-F	GGTGGTGCTTTGTTGATGATATT	RT-qPCR primer
PSYR1-RT-R	TCGTCTCGTCTATGTCCTTCT	RT-qPCR primer
PSYR2-RT-F	GCCTCTTTGCGAAGCTAAATG	RT-qPCR primer
PSYR2-RT-R	AGCCATCGGTGTTGGTAAG	RT-qPCR primer
PSY-RT-F	TTCCAGCAAGAGAAGGTTGG	RT-qPCR primer
PSY-RT-R	CTCCACGAGATTTGCCTCAG	RT-qPCR primer
Pp3c2_1870-RT-F	GCGCGAGTGGTAGTAATGAA	RT-qPCR primer
Pp3c2_1870-RT-R	ACTGATCTGCGGGCTTTAAT	RT-qPCR primer
Pp3c3_36680-RT-F	GATGTTAAGGCTGCCAACATTC	RT-qPCR primer
Pp3c3_36680-RT-R	GTGACATGGGTGTCCTTATAGTC	RT-qPCR primer
PpERF138-RT-F	AATGGCAGCTAGAGCGTATG	RT-qPCR primer

PpERF138-RT-R	AGTGGCATTCAGGAAGTGATT	RT-qPCR primer
Pp3c9_1620-RT-F	CACGAATACCGACCGACTTT	RT-qPCR primer
Pp3c9_1620-RT-R	CCTCGTCATAGAACGTGAACTC	RT-qPCR primer
