# Supplementary material for: The Sulfated PSY Peptide Negatively Regulates Receptor Kinase Activity to Promote Growth
Source: bioRxiv. 2026 Sep 2:2026.07.25.740709. Preprint. [Version 2] doi: 10.64898/2026.07.25.740709 (PMC13618415; doi:10.64898/2026.07.25.740709)
Supplement: Supplementary file 4 [file media-2.pdf]

A *PpPSYR1*

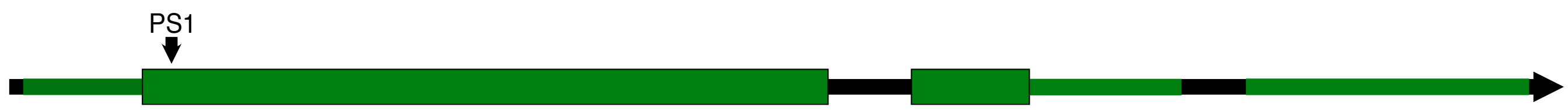

Protospacer 1 19 TTGCTCGGGTGGGCTCTCGCCGTGAGCTTAGTGGCGTTGCTG 32
L L G W A L A V S L V A L L

Δpsyr2/Δpsyr1-14 *(Δpsyr1/2)* TTGCTCGGGTGGGCTCTCGCCGTG----------GCGTTGCTG (Stop aa 41)
L L G W A L A V A L L

33 TCGTGTCGGAGTTGCTGTGGGCTGTCCCCCAACTGA 41
S C R S C C G L S P N *

B *PpPSYR2*

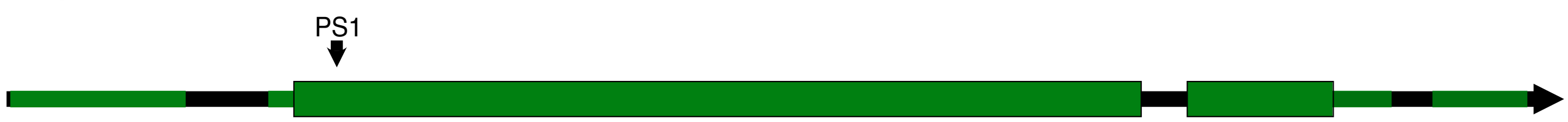

Protospacer 1 104 GGCAAGCTTGCGGCCCTCCGGTATCTTAACCTGTCGTCGAAC 117
G K L A A L R Y L N L S S N

Δpsyr1/2 GGTTAAGCTT------------------AACCTGTCGTCGTA (Stop aa 105)
G *

C

PpTPST

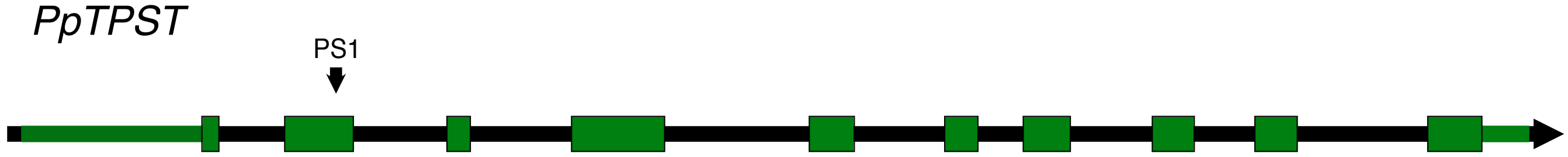

Protospacer 1 74 CTGTTCTTTTTGCATATTCCTCGAACCGGCGGCCGGACTTAT 87
L F F L H I P R T G G R T Y

Δpsyr1/2/Δtpst-3, *(Δpsyr1/2/Δtpst)* CTGTTCTTTTTGCATATTCCTCGAATAGCTGATTAAACTTAT (Stop aa 85)
L F F L H I P R I A D *

Figure S2
