# Supplementary material for: The Sulfated PSY Peptide Negatively Regulates Receptor Kinase Activity to Promote Growth
Source: bioRxiv. 2026 Sep 2:2026.07.25.740709. Preprint. [Version 2] doi: 10.64898/2026.07.25.740709 (PMC13618415; doi:10.64898/2026.07.25.740709)
Supplement: Supplementary file 5 [file media-3.pdf]

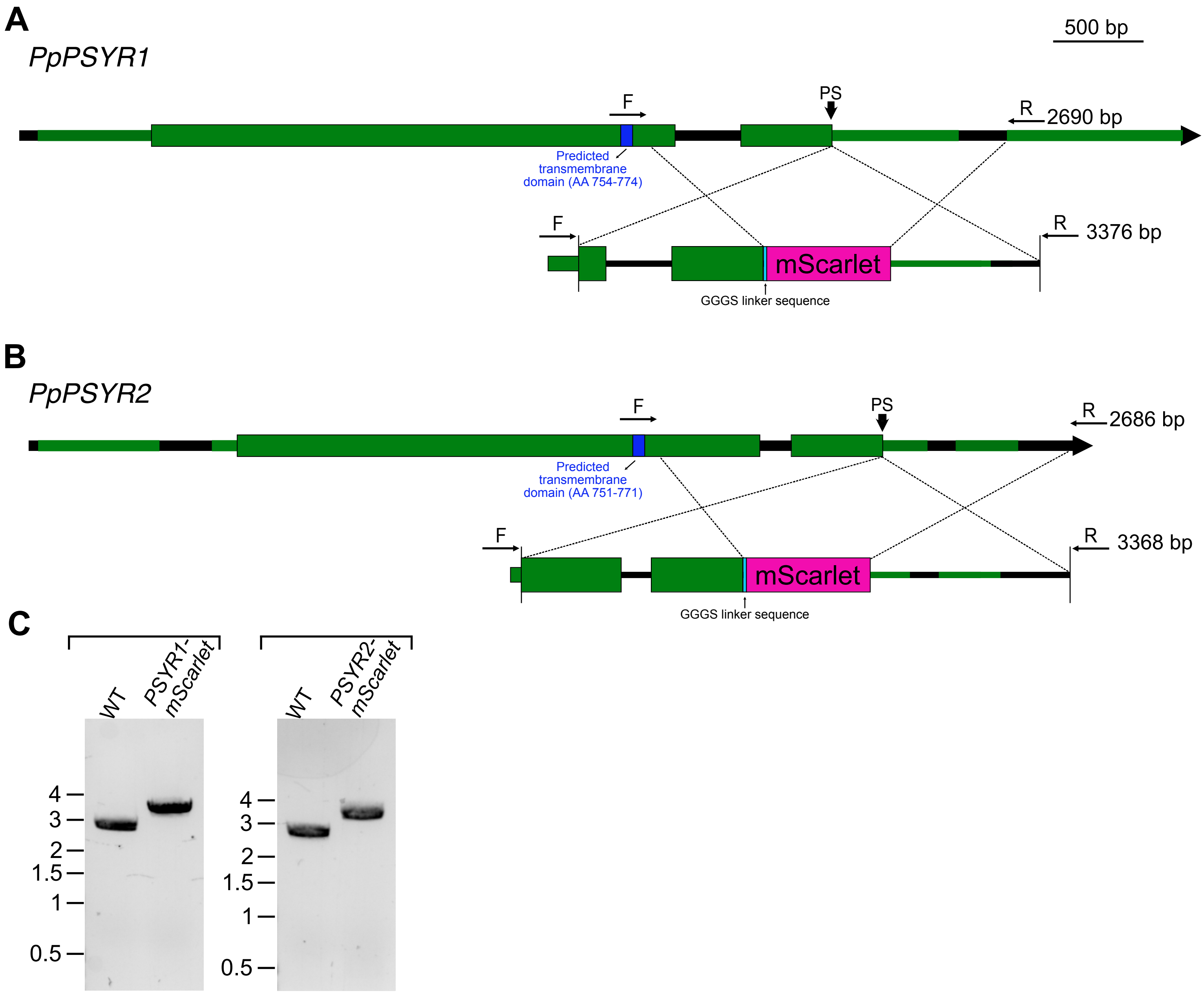

A
PpPSYR1
500 bp
F
PS
R
2690 bp
Predicted transmembrane domain (AA 754-774)
F
R
3376 bp
mScarlet
GGGS linker sequence
B
PpPSYR2
F
PS
R
2686 bp
Predicted transmembrane domain (AA 751-771)
F
R
3368 bp
mScarlet
GGGS linker sequence
C
WT
PSYR1-mScarlet
WT
PSYR2-mScarlet
4
3
2
1.5
1
0.5
4
3
2
1.5
1
0.5

Figure S3
