# Supplementary material for: The Sulfated PSY Peptide Negatively Regulates Receptor Kinase Activity to Promote Growth
Source: bioRxiv. 2026 Sep 2:2026.07.25.740709. Preprint. [Version 2] doi: 10.64898/2026.07.25.740709 (PMC13618415; doi:10.64898/2026.07.25.740709)
Supplement: Supplementary file 6 [file media-4.pdf]

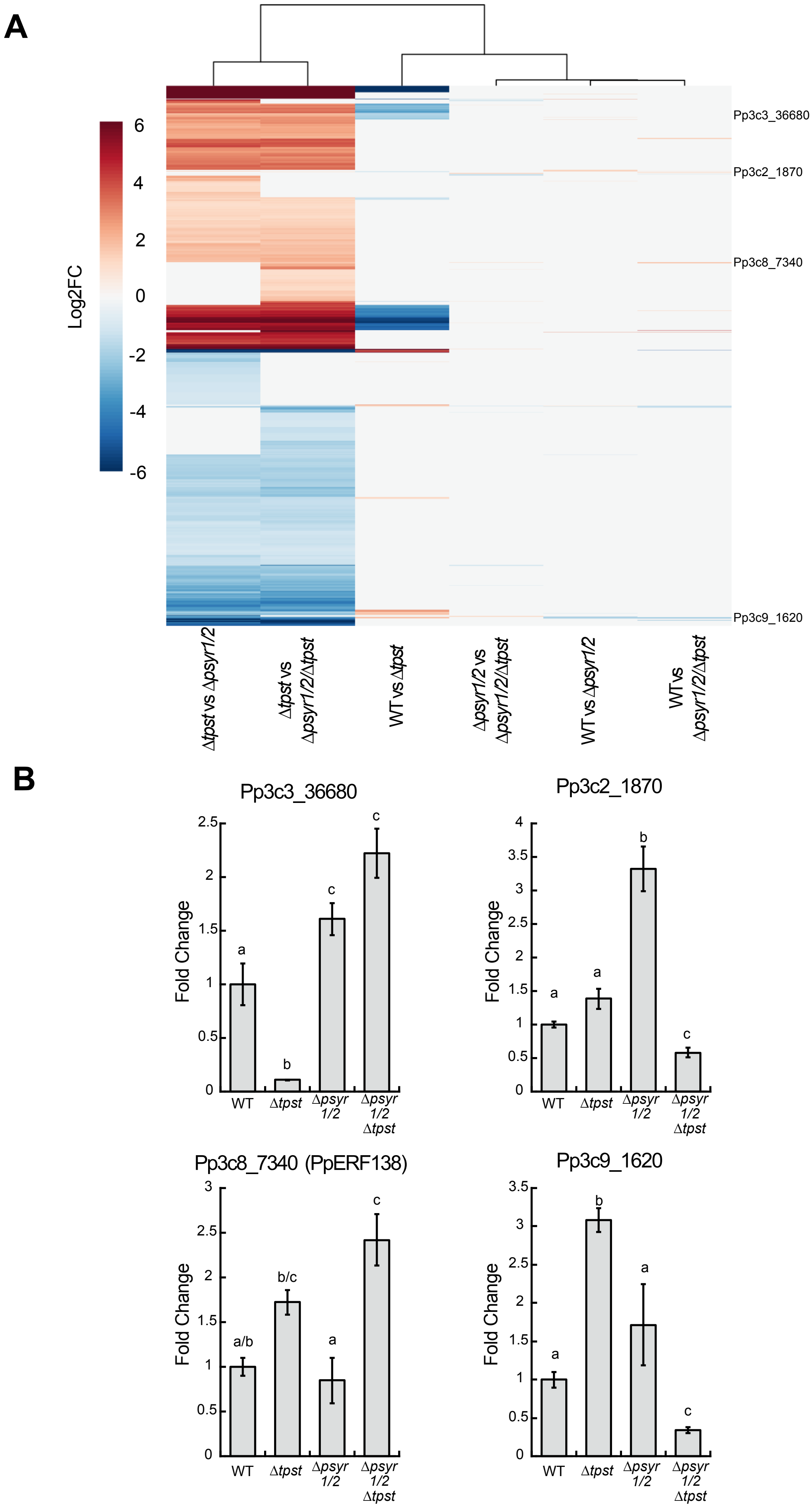
A
Log2FC
6
4
2
0
-2
-4
-6
Pp3c3_36680
Pp3c2_1870
Pp3c8_7340
Pp3c9_1620
Δtpst vs Δpsyr1/2
Δtpst vs Δpsyr1/2/Δtpst
WT vs Δtpst
Δpsyr1/2 vs Δpsyr1/2/Δtpst
WT vs Δpsyr1/2
WT vs Δpsyr1/2/Δtpst
B
Pp3c3_36680
Pp3c2_1870
Pp3c8_7340 (PpERF138)
Pp3c9_1620
Fold Change
WT
Δtpst
Δpsyr 1/2
Δpsyr 1/2 Δtpst

Figure S4
