# Supplementary material for: The Sulfated PSY Peptide Negatively Regulates Receptor Kinase Activity to Promote Growth
Source: bioRxiv. 2026 Sep 2:2026.07.25.740709. Preprint. [Version 2] doi: 10.64898/2026.07.25.740709 (PMC13618415; doi:10.64898/2026.07.25.740709)
Supplement: Supplementary file 7 [file media-5.pdf]

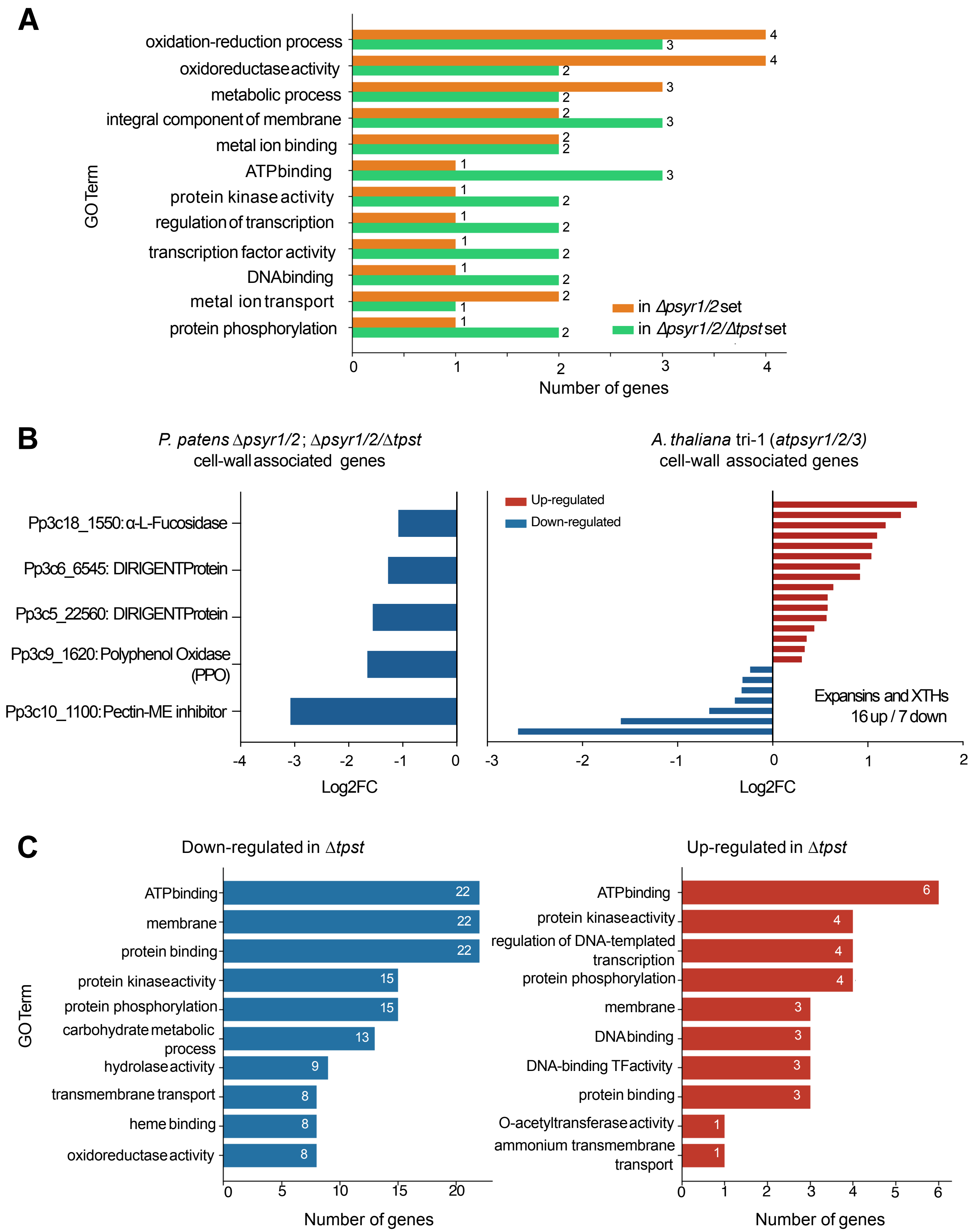

A
GO Term
oxidation-reduction process
oxidoreductase activity
metabolic process
integral component of membrane
metal ion binding
ATP binding
protein kinase activity
regulation of transcription
transcription factor activity
DNA binding
metal ion transport
protein phosphorylation
in Δpsyr1/2 set
in Δpsyr1/2/Δtpst set
Number of genes
B
P. patens Δpsyr1/2 ; Δpsyr1/2/Δtpst
cell-wall associated genes
A. thaliana tri-1 (atpsyr1/2/3)
cell-wall associated genes
Pp3c18_1550: α-L-Fucosidase
Pp3c6_6545: DIRIGENT Protein
Pp3c5_22560: DIRIGENT Protein
Pp3c9_1620: Polyphenol Oxidase (PPO)
Pp3c10_1100: Pectin-ME inhibitor
Up-regulated
Down-regulated
Expansins and XTHs
16 up / 7 down
Log2FC
C
Down-regulated in Δtpst
Up-regulated in Δtpst
GO Term
ATP binding
membrane
protein binding
protein kinase activity
protein phosphorylation
carbohydrate metabolic process
hydrolase activity
transmembrane transport
heme binding
oxidoreductase activity
regulation of DNA-templated transcription
DNA binding
DNA-binding TF activity
O-acetyltransferase activity
ammonium transmembrane transport
Number of genes

Figure S5
