# Supplementary material for: The Sulfated PSY Peptide Negatively Regulates Receptor Kinase Activity to Promote Growth
Source: bioRxiv. 2026 Sep 2:2026.07.25.740709. Preprint. [Version 2] doi: 10.64898/2026.07.25.740709 (PMC13618415; doi:10.64898/2026.07.25.740709)
Supplement: Supplementary file 8 [file media-6.pdf]

A

```
At_BAK1   RKKPQDHFFDVPAEEDPEVHLGQLKRFSLRELQVASDNFSNKNILGRGGFGKVYKGRLAD  310
Pp_PSYR1  RRPPSARQVASEKDIDETIFLPR-AGVTLQDIVTATENFSDEKVIGKGACGTVYKAQMPG  836
Pp_PSYR2  RRPPGATQVASEKDMDETIFLPR-TGVSLQDIIAATENFSNTKVIGKGASGTVYKAVMVS  834

At_BAK1   GTLVAVKRLKEERTQGGEL--QFQTEVEMISMAVHRNLLRLRGFCMTPTERLLVYPYMAN  368
Pp_PSYR1  GQLIAVKKVATHLDSGLTQHDSFTAEIKTLGKIRHRNIVKLLGFCSYQGYNLLMYDYMPK  896
Pp_PSYR2  GQVIAVKKMSTQTESGLTQIDSFTAEIKTLGKIRHRNIVKLLGFCSYQGCNLLMYDYMPK  894

At_BAK1   GSVASCLRERPESQPPLDWPKRQRIALGSARGLAYLHDHCDPKIIHRDVKAANILLDEEF  428
Pp_PSYR1  GSLGEHLVKK---DCELDWDLRYKIAVGSAEGLEYLHHDCKPLIIHRDIKSNNILLNERY  953
Pp_PSYR2  GSLGDLLAKE---DCELDWDLRYKIAVGSAEGLEYLHHDCKPLILHRDIKSTNILLDDHF  951

At_BAK1   EAVVGDFGLAKLMDYKDTHVTTAVRGTIGHIAPEYLSTGKSSEKTDVFGYGVMLLELITG  488
Pp_PSYR1  EAHVGDFGLAKLIDLAETKSMSAIAGSYGYIAPEYAYTMNVTEKSDIYSFGVVLLELLTG  1013
Pp_PSYR2  KAHVGDFGLAKLFDFADTKSMSAIAGSYGYIAPEYAYTMNVTEKSDIYSFGVVLLELLTG  1011

At_BAK1   QRAFDLARLANDDDVMLLDWVKGLLK-EKKLEALVDV--DLQGNYKDEEVEQLIQVALLC  545
Pp_PSYR1  RRPIQPV----DEGGDLVTWVKEAMQLHKSVSRIFDIRLDLTDVVIIEEMLLVLRVALFC  1069
Pp_PSYR2  RHPIQHI----DDGGDLVTWVKEAMQLHRSVSRIFDTRLDLTDVVIIEEMLLVLKVALFC  1067

At_BAK1   TQSSPMERPKMSEVVRMLEGDGLAERWE--EWQKEEMFRQDFNYP---------THHPAV  594
Pp_PSYR1  TSSLPQERPTMREVVRMLMEASTRKARDSTDSQSETQGRESVSDGTITDGTLTIADDTLT  1129
Pp_PSYR2  TSSLPQERPTMREVVRMLMEASTRKARDSTDLQSET---QDACENGDSVSDAAVTRDTVI  1124
```

B *PpPSYR1*

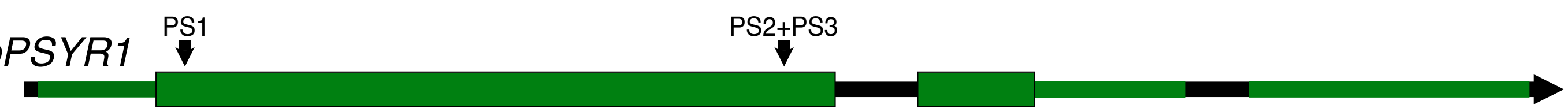


```
Protospacer 2  833  CAAATGCCTGGCGGTCAGCTAATAGCGGTCAAGAAGGTTGCG  845
                    Q  M  P  G  G  Q  L  I  A  V  K  K  V  A

Protospacer 3  833  CAAATGCCTGGCGGTCAGCTAATAGCGGTCAAGAAGGTTGCG  845
                    Q  M  P  G  G  Q  L  I  A  V  K  K  V  A
```

PSYR1-mScarlet/ PSYR1-K843E/Δpsyr2 (psyr1-KD)

```
CAAATGCCTGGaGGaCAatTgATtGCtGTCgAGAAGGTTGCG
Q  M  P  G  G  Q  L  I  A  V  E  K  V  A
```

Δtpst/ PSYR1-mScarlet/ PSYR1-K843E/Δpsyr2 (Δtpst/psyr1-KD)

```
CAAATGCCTGGaGGaCAattgATtGCtGTCgAGAAGGTTGCG
Q  M  P  G  G  Q  L  I  A  V  E  K  V  A
```

C *PpPSYR2*

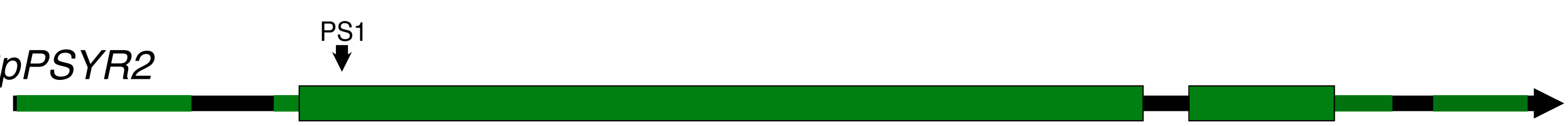


```
Protospacer 1  104  GGCAAGCTTGCGGCCCTCCGGTATCTTAACCTGTCGTCGAACCGGCTGACC  120
                    G  K  L  A  A  L  R  Y  L  N  L  S  S  N  R  L  T
```

PSYR1-mScarlet/ PSYR1-K843E/Δpsyr2 (psyr1-KD)

```
GACAAGTGTGGGATTT---------------GTATCTTAACCTGTCGTCGAACCGGCTGA   (Stop aa 118)
D  K  C  G  I  C                   I  L  T  C  R  R  T  G  *
```

Δtpst/ PSYR1-mScarlet/ PSYR1-K843E/Δpsyr2 (Δtpst/psyr1-KD)

```
GGCAAGCTTGCGGC-------TATCTTAACCTGTCGTCGAACCGGCTGACC   (Stop aa 117)
G  K  L  A  A        I  L  T  C  R  R  T  G  *
```

Figure S6
