# Supplementary material for: The Sulfated PSY Peptide Negatively Regulates Receptor Kinase Activity to Promote Growth
Source: bioRxiv. 2026 Sep 2:2026.07.25.740709. Preprint. [Version 2] doi: 10.64898/2026.07.25.740709 (PMC13618415; doi:10.64898/2026.07.25.740709)
Supplement: Supplementary file 10 [file media-8.pdf]

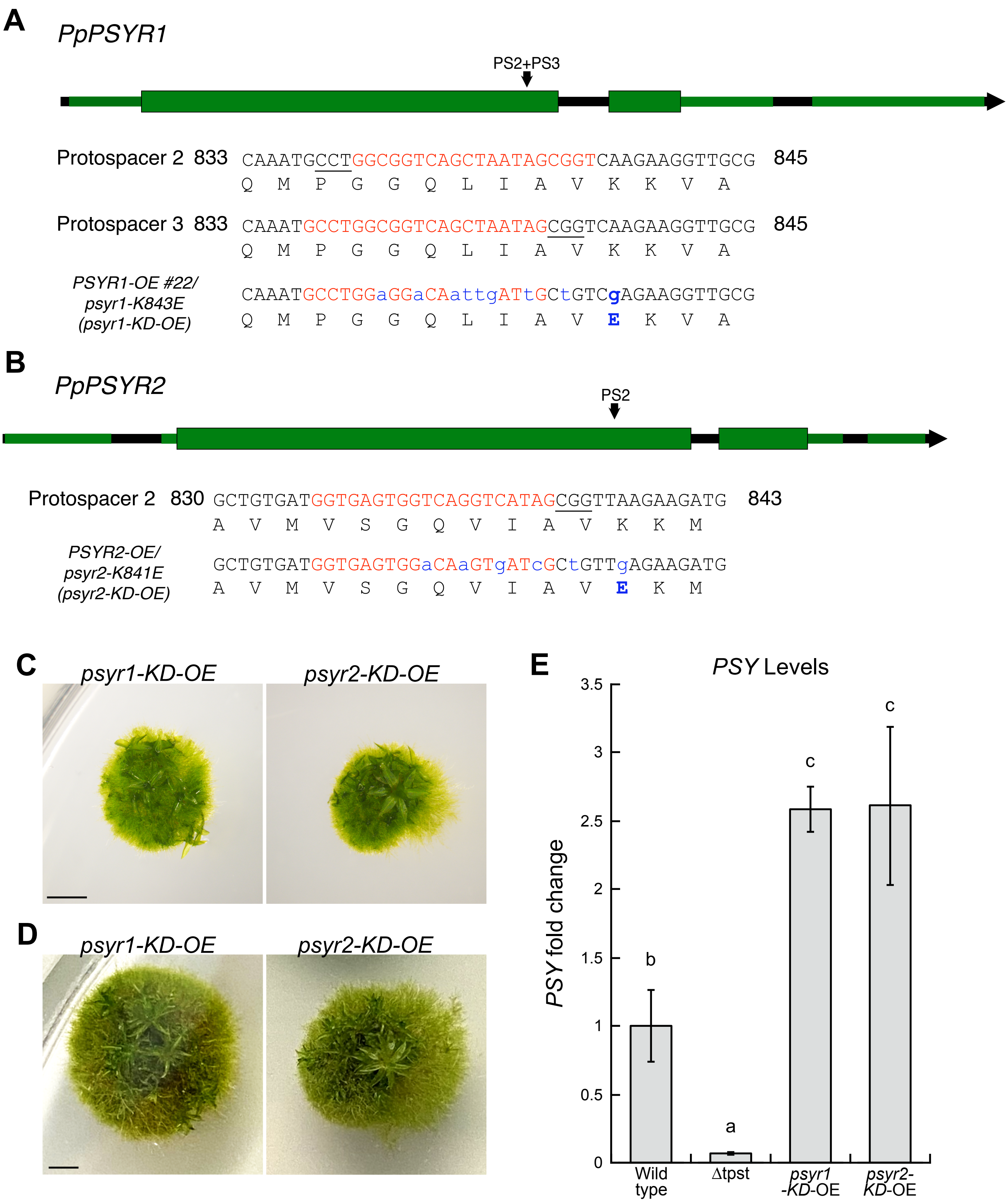
A
PpPSYR1
PS2+PS3
Protospacer 2 833 CAAATGCCTGGCGGTCAGCTAATAGCGGTCAAGAAGGTTGCG 845
Q M P G G Q L I A V K K V A
Protospacer 3 833 CAAATGCCTGGCGGTCAGCTAATAGCGGTCAAGAAGGTTGCG 845
Q M P G G Q L I A V K K V A
PSYR1-OE #22/ psyr1-K843E (psyr1-KD-OE)
CAAATGCCTGGaGGaCAattgATtGCtGTCgAGAAGGTTGCG
Q M P G G Q L I A V E K V A
B
PpPSYR2
PS2
Protospacer 2 830 GCTGTGATGGTGAGTGGTCAGGTCATAGCGGTTAAGAAGATG 843
A V M V S G Q V I A V K K M
PSYR2-OE/ psyr2-K841E (psyr2-KD-OE)
GCTGTGATGGTGAGTGGaCAaGTgATcGCtGTTgAGAAGATG
A V M V S G Q V I A V E K M
C
psyr1-KD-OE
psyr2-KD-OE
D
psyr1-KD-OE
psyr2-KD-OE
E
PSY Levels
PSY fold change
3.5
3
2.5
2
1.5
1
0.5
0
b
a
c
c
Wild type
Δtpst
psyr1 -KD-OE
psyr2-KD-OE

Figure S8
